# A Case of Plasmodium vivax-Induced Stress Cardiomyopathy Managed With Extracorporeal Membrane Oxygenation

**DOI:** 10.7759/cureus.84412

**Published:** 2025-05-19

**Authors:** Diane Dreucean, Jesse E Harris, Hala Halawi, Alejandro Granillo, Jose F Cuevas

**Affiliations:** 1 Pharmacy, Houston Methodist Hospital, Houston, USA; 2 Infectious Diseases, Houston Methodist Hospital, Houston, USA; 3 Anesthesiology and Critical Care, Houston Methodist Hospital, Houston, USA

**Keywords:** ards (acute respiratory distress syndrome), plasmodium vivax malaria, short term mechanical circulatory support, stress cardiomyopathy, takostubo cardiomyopathy, veno-arterial ecmo, vv ecmo

## Abstract

Acute respiratory distress syndrome (ARDS) and cardiomyopathy can occur as severe manifestations of malarial infections. We present a case of a 20-year-old male who, after recently returning from Nigeria, presented to our hospital with flu-like symptoms and was ultimately diagnosed with *Plasmodium vivax *infection. He developed severe ARDS and failed conservative management with lung-protective ventilation, steroids, and inhaled pulmonary vasodilators. Given the severity of his disease, he was placed on venovenous extracorporeal membrane oxygenation (ECMO) to provide further pulmonary support. He subsequently developed acute stress cardiomyopathy with severe biventricular failure that did not respond to high-dose vasopressor and inotropic therapy. His cannula configuration was adjusted to veno-arterial-venous (VAV) ECMO to provide additional hemodynamic support. During this time, the patient had been receiving treatment with artesunate. After a total of seven days on ECMO, he was successfully decannulated and safely discharged home with oral antimalarial treatment.

## Introduction

Patients with malarial infections can develop life-threatening complications such as pulmonary edema, malaria-associated acute respiratory distress syndrome (MA-ARDS), and cardiomyopathies [1-4]. Previous case reports have documented the novel use of venovenous extracorporeal membrane oxygenation (VV ECMO) to support pulmonary recovery in patients with severe ARDS [5]. While approximately 14% of patients develop severe manifestations, there is a paucity of data outlining treatment modalities of cardiovascular complications [2]. The majority of cardiac complications with Plasmodium species infections include myocarditis and pericarditis, as well as acute heart failure, for which treatment consists of antimalarial agents to treat the underlying etiology [2]. The majority of cardiac complications described in the literature exist with *Plasmodium falciparum* infections, with scant data regarding incidence and outcomes regarding* Plasmodium vivax*-related cardiomyopathies [2]. This report outlines veno-arterial-venous ECMO (VAV ECMO) in a patient with MA-ARDS and stress cardiomyopathy due to severe *Plasmodium vivax *infection.

## Case presentation

A 20-year-old man with a past medical history of asthma presented to the emergency department with a three-day history of flu-like symptoms. Six days before this encounter, the patient returned to the United States after ten days in Nigeria; he did not use antimalarial prophylaxis before or during this trip. He presented as ill-appearing, afebrile, tachycardic, hypertensive, hypoxic, and in respiratory distress. He was placed on noninvasive positive pressure ventilation with improvement in oxygenation. Admission bloodwork was notable for serum creatinine 1.18 mg/dL, troponin T 33 ng/L, alanine transaminase (ALT) 144 units/L, aspartate transaminase (AST) 135 units/L, white blood cell count 8,600 cells/microliter, platelet count 35,000 cells/microliter, and normal hemoglobin at 15.3 g/dL (Table 1). An arterial blood gas (ABG) demonstrated pH 7.38, partial pressure of carbon dioxide (pCO_2_) 39 mmHg, pO_2_ 74 mmHg, and arterial oxygen saturation (SaO_2_) 94%. A chest X-ray showed multifocal infiltrates consistent with pneumonia; CTA chest was negative for pulmonary embolism. Additional testing, including blood and sputum cultures, respiratory pathogen polymerase chain reaction (PCR) panel, rickettsia serologies, and peripheral smear to evaluate for the presence of trophozoites, was obtained. The patient was started on empirical therapy with vancomycin, meropenem, and doxycycline. 

**Table 1:** Laboratory Values ALT: alanine transaminase; AST: aspartate transaminase; WBC: white blood cell; pCO2: partial pressure of carbon dioxide; pO2: partial pressure of oxygen; SaO2: arterial oxygen saturation; G6PD: glucose 6-phosphate dehydrogenase; VV ECMO: venovenous extracorporeal membrane oxygenation; VAV ECMO: veno-arterial-venous extracorporeal membrane oxygenation.

Lab	Values	Normal Range
Labs on admission		
Creatinine (mg/dL)	1.18	0.50 – 0.90
Troponin T (ng/L)	33	0 – 19
ALT (units/L)	144	50 – 100
AST (units/L)	135	10 – 35
WBC count (cells/mcL)	8,600	4,500 – 11,000
Platelet count (cells/mcL)	35,000	150,000 – 400,000
Hemoglobin (g/dL)	15.3	12.0 – 16.0
pH, arterial	7.38	7.3 – 7.45
pCO2, arterial (mmHg)	39	35 – 45
pO2, arterial (mmHg)	74	80 – 90
SaO2, arterial (%)	94	95 – 100
G6PD, quantitative (units/g Hb)	5.8	9.9 – 16.6
Labs at 6 hours		
pH, arterial	7.30	7.3 – 7.45
pCO2, arterial (mmHg)	43	35 – 45
pO2, arterial (mmHg)	57	80 – 90
SaO2, arterial (%)	83	95 – 100
Labs post-VV ECMO cannulation		
pH, arterial	7.37	7.3 – 7.45
pCO2, arterial (mmHg)	30	35 – 45
pO2, arterial (mmHg)	204	80 – 90
SaO2, arterial (%)	99	95 – 100
Labs post-reconfiguration to VAV ECMO		
pH, arterial	7.36	7.3 – 7.45
pCO2, arterial (mmHg)	45	35 – 45
pO2, arterial (mmHg)	58	80 – 90

Within six hours of admission, the patient developed progressive hypoxemia. A repeat ABG showed pH 7.30, pCO_2_ 43 mmHg, pO_2_ 57 mmHg, and SaO_2_ 83% (Table 1). He was subsequently intubated, but he remained profoundly hypoxic with SpO_2_ readings ranging from 80-84%. Attempts to escalate ventilatory support were unsuccessful. Neuromuscular junction blockade and inhaled epoprostenol were added with minimal improvement in oxygenation. Consequently, after 8 hours from presentation, the patient was cannulated onto VV ECMO via a 20 French right internal jugular (IJ) venous cannula and a 25 French right femoral vein (RFV) venous cannula. The initial ECMO settings included a flow of 3.1 liters per minute (LPM), sweep gas flow (SGF) rate of 2 LPM, and 100% pump FiO2. Anticoagulation was started with bivalirudin.

Following cannulation, a repeat ABG showed overall improvement in oxygenation with pH 7.37, pCO_2_ 30 mmHg, pO_2_ 204 mmHg, and SaO_2_ 99% (Table 1). Ventilator settings were adjusted to assist-control volume mode with a tidal volume of 5.5 mL/kg, 100% FiO2, and positive end expiratory pressure (PEEP) of 14 mmHg. Approximately eight hours after cannulation, a thin blood smear revealed *Plasmodium vivax* with 1% parasitemia, and the patient was subsequently started on intravenous artesunate 220 mg (2.2 mg/kg) twice daily. Additionally, the patient developed hemodynamic instability with declining mean arterial pressures (MAP) to 50 to 55 mmHg. Norepinephrine and vasopressin were initiated to maintain a MAP of 65 mmHg. A transthoracic echocardiogram (TTE) demonstrated a left ventricular ejection fraction (LVEF) of 50% with normal wall motion and normal right ventricular (RV) size.

**Figure 1:**
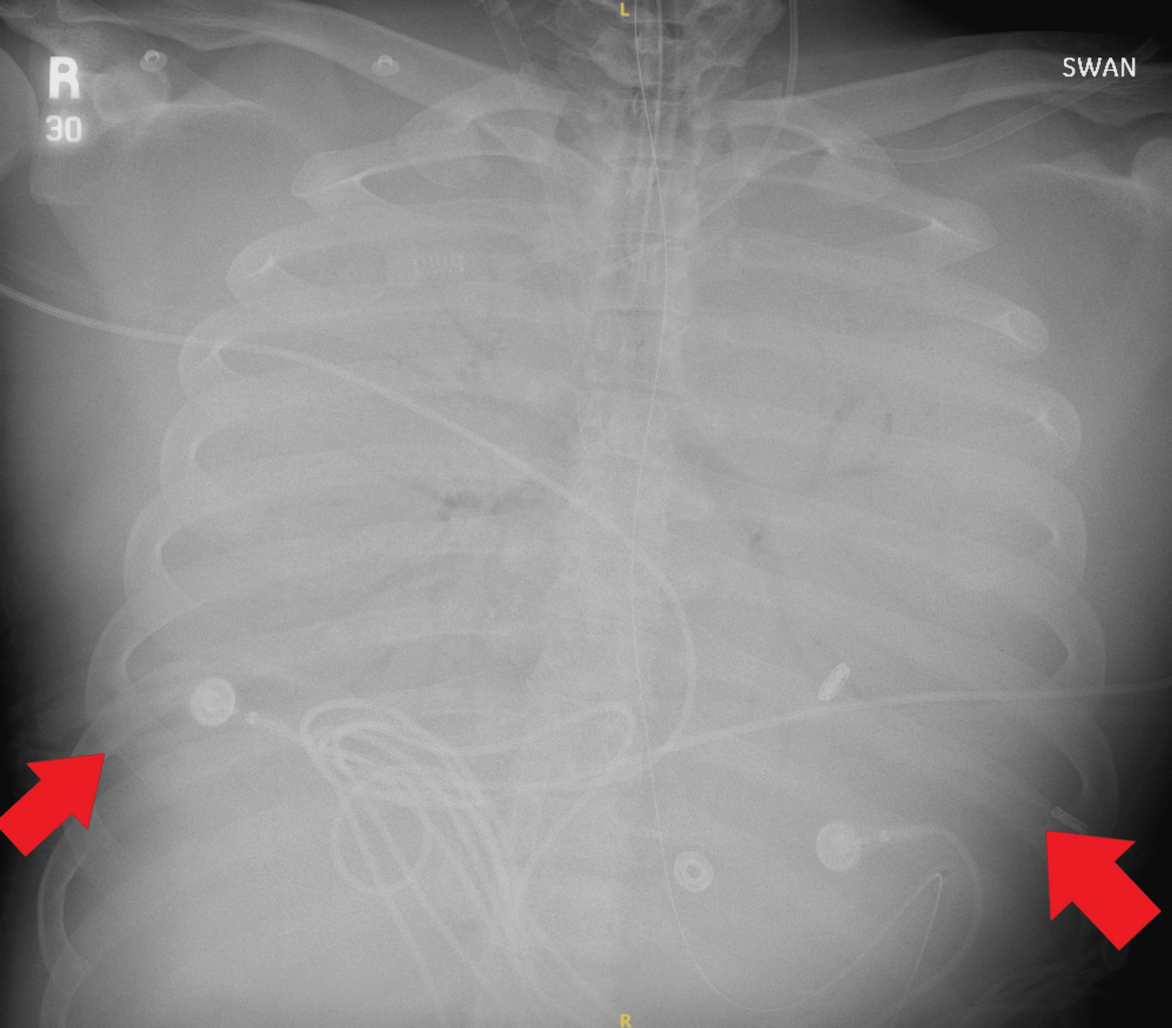
Bilateral Lung Whiteout

**Figure 2:**
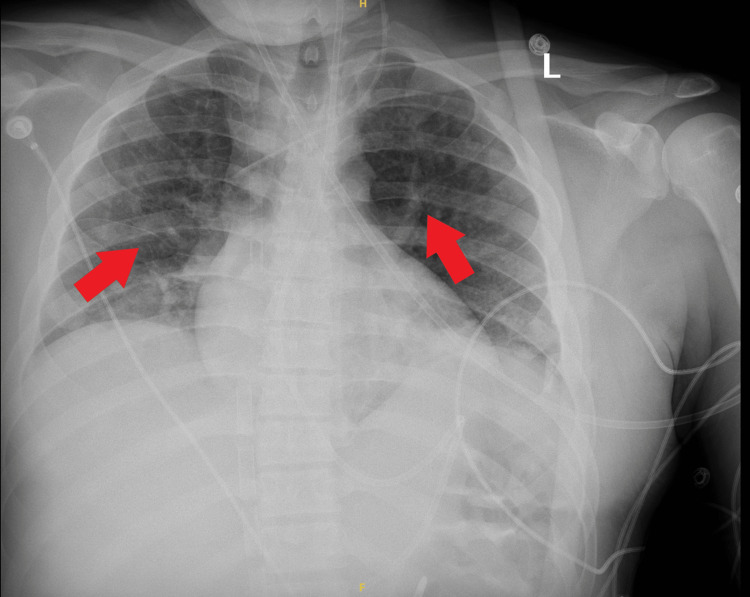
Improved Alveolar Recruitment After APRV APRV: airway pressure release ventilation

Given persistently high vasopressor support with norepinephrine 12 mcg/min and vasopressin 0.04 units/min, a repeat TTE was performed six hours later, which revealed severe biventricular dysfunction with LVEF 25%, global hypokinesis, and enlarged and severely depressed RV. Dobutamine was initiated with minimal improvement in hemodynamics, and lactate continued to climb up to 6.6 mmol/L. An electrocardiogram (ECG) was done, which showed no major abnormalities. Due to renal failure, continuous renal replacement therapy (CRRT) was initiated. Given the continued decline in hemodynamics, the patient was escalated to VAV ECMO using a 19 French return cannula in the right common femoral artery, which was Y-sited to the right IJ return cannula. The RFV cannula was retained to provide drainage from the inferior vena cava. Following cannulation reconfiguration, the ECMO settings were adjusted to a flow of 5.2 LPM, SGF 6 LPM, and 100% pump FiO2. Repeat ABG demonstrated pH 7.36, pCO_2_ 45 mmHg, and pO_2_ 58 mmHg (Table 1). Despite increases in ECMO flows, pO2 did not significantly improve. The arterial return cannula was clamped to divert more flow to the venous return port, but this did not yield any significant benefit. A chest x-ray demonstrated bilateral lung whiteout, thus increasing suspicion for intrapulmonary shunting as the cause of persistent hypoxemia (Figure 1). A repeat TTE showed persistent severe biventricular dysfunction, spontaneous contrast in the LV, and a reverse-takotsubo pattern of akinesis of the basal and mid-segments with hypokinesis of the apex (Video 1). The ventilator was subsequently adjusted to airway pressure release ventilation (APRV) mode with P_high_ 30 cm H2O, and inhaled nitric oxide was initiated at 40 parts per million (ppm). After 24 hours, oxygenation began to improve. Serial chest x-rays also demonstrated improvement in bilateral lung whiteout consistent with improved lung alveolar recruitment (Figure 2).

**Video 1:** Reverse-Takotsubo Pattern of Akenesis

Over the next 36 hours, the patient’s oxygenation continued to improve significantly. A repeat TTE demonstrated normalization of biventricular function (Video 2). The arterial ECMO cannula was removed, and the patient was transitioned back to VV ECMO. The pending infectious workup was negative, and antibacterials were de-escalated. Over the next three days, VV ECMO was weaned, and the patient was successfully decannulated from all ECMO support and liberated from the ventilator without complications. The total duration of ECMO support was seven days.

**Video 2:** Normalization of Biventricular Function

Significant clinical improvement was observed, and the patient was transferred out of the intensive care unit (ICU) to a regular acute care unit. He completed a total of seven days of intravenous (IV) artesunate therapy followed by three days of artemether/lumefantrine. Of note, he was found to have glucose-6-phosphate dehydrogenase (G6PD) deficient at 5.8 units/g Hb (normal 9.9-16.6 units/g Hb). Given the above enzymatic deficiency and the need for continued therapy to prevent relapses in the setting of possible hepatic hypnozoites with *Plasmodium vivax* infections, he was prescribed hydroxychloroquine 400 mg weekly for one year, with close outpatient monitoring. After a total hospitalization of 14 days, he was successfully discharged home.

## Discussion

While most malaria infections in adults do not cause major complications, MA-ARDS and cardiomyopathies remain serious complications that can occur. Most MA-ARDS cases have occurred in the setting of *Plasmodium falciparum* infections. However, severe ARDS has also been observed with *Plasmodium vivax* [4]. Similar trends are seen in patients with cardiac complications from *Plasmodium* species. We present a case of *Plasmodium vivax* parasitemia complicated by ARDS and stress cardiomyopathy that was successfully managed using VAV ECMO.

Malaria-induced cardiovascular complications have been previously described in the literature and primarily include myocarditis and acute coronary syndromes (ACS) [3]. Several pro-inflammatory mediators have been implicated in the development of cardiomyopathies from Plasmodium infections. The overwhelming release of cytokines can lead to erythrocytic rupture, nitric oxide-induced energy depletion, and direct cardiac cell apoptosis due to the release of plasmodial glycosylphosphatidiylinositol [2]. Secondary effects from hemolytic anemia can also contribute to myocardial ischemia and eccentric left ventricular remodeling. Though our patient did not have significant anemia on initial presentation, this could be explained by hemoconcentration related to intravascular depletion. His hemoglobin nadir was at 6.6 g/dL during the hospitalization. Additionally, acute renal failure and subsequent volume overload may also worsen cardiac function and hemodynamics [2]. Finally, antimalarial drugs used to treat acute infections have been associated with severe cardiac side effects; however, these are mainly QTc prolongation in nature, and artesunate has not been associated with these effects. Upon presentation, our patient exhibited normal cardiac function, but he rapidly progressed to developing acute biventricular failure and ultimately cardiogenic shock, demonstrated by increasing vasopressor requirements and signs of hypoperfusion, including lactic acidosis. Given the involvement of both ventricles along with no specific ST segment changes on ECG, ACS was ruled out. Myocarditis was also initially considered as part of the differential, and therefore, intravenous corticosteroids were administered; however, the lack of any significant elevations in troponin and creatine kinase throughout his illness suggested stress cardiomyopathy as the likely underlying etiology. Historically, the level of parasitemia has been a risk factor for infection severity, with higher levels corresponding to more invasive disease. Infections with *Plasmodium vivax*, however, have demonstrated severe complications despite lower levels of parasitemia. This suggests that *Plasmodium vivax *may be responsible for a heightened inflammatory response [3,4]. This is consistent with our patient’s clinical presentation of *Plasmodium vivax* with 1% parasitemia progressing to severe cardiopulmonary failure, necessitating the use of ECMO support. 

Currently, targeted therapies for the management of stress cardiomyopathy are lacking. General management primarily focuses on the use of guideline-directed medical therapy and supportive care. In our patient, the presence of hemodynamic instability and acute renal failure limited the use of these therapies. It is unclear whether the stress cardiomyopathy in our patient was a direct consequence of the plasmodium infection or if it occurred secondary to ARDS and subsequent hypoxia. Nonetheless, irrespective of the insult, the management approach would involve general hemodynamic support. As such, the patient was supported with the addition of an arterial cannula to his VV ECMO configuration to ensure adequate perfusion and allow for myocardial recovery. Anticoagulation has also been recommended in patients with stress cardiomyopathy accompanied by acute left ventricular dysfunction to prevent LV thrombus formation. In our case, we chose to utilize bivalirudin over unfractionated heparin, considering the patient’s ongoing thrombocytopenia and given the potential benefits of bivalirudin in patients with thrombocytopenia receiving ECMO support.

Overall, our patient was well supported on VAV ECMO following the development of cardiogenic shock, and ultimately was discharged home with cardiology and infectious diseases follow-up to ensure close monitoring of myocardial recovery. He has remained free of any subsequent needs for hospitalization following his initial admission, demonstrating the reversibility of his myocardial dysfunction with antimalarial treatment. 

## Conclusions

To the best of our knowledge, this is the first documented case of *Plasmodium vivax* infection leading to stress cardiomyopathy that was managed using VAV ECMO. Outcomes related to hemodynamic support with ECMO in patients with malaria-associated cardiac and pulmonary dysfunction remain widely unknown; however, our patient recovered quickly after initiation of anti-malarial therapy, which may demonstrate the benefit of early initiation of temporary mechanical circulatory support to allow for lung rest and recovery and hemodynamic decline. While severe complications from malarial infections remain rare, providers must be aware of the possible management strategies and outcomes associated with the use of mechanical circulatory support in this population. Long-term follow-up for monitoring of cardiopulmonary recovery and monitoring for malaria relapse is needed with this high-risk patient population. Given the small sample size and inability to conclude a single case, further studies are needed to investigate the role and timing of ECMO initiation as a bridge to recovery in this patient population.
